# Correction: The robust estimation of examinee ability based on the four-parameter logistic model when guessing and carelessness responses exist

**DOI:** 10.1371/journal.pone.0258023

**Published:** 2021-09-23

**Authors:** Xiaozhu Jian, Buyun Dai, Yuanping Deng

The second and third authors’ names are incorrect. The correct name for the second author is Buyun Dai and the correct name for the third author is Yuanping Deng.

The correct citation is: Jian X, Dai B, Deng Y (2021) The robust estimation of examinee ability based on the four-parameter logistic model when guessing and carelessness responses exist. PLoS ONE 16(4): e0250268. https://doi.org/10.1371/journal.pone.0250268
