# Supplementary material for: The robust estimation of examinee ability based on the four-parameter logistic model when guessing and carelessness responses exist
Source: PLoS One. 2021 Apr 29;16(4):e0250268. doi: 10.1371/journal.pone.0250268 (PMC8084210; doi:10.1371/journal.pone.0250268)
Supplement: S1 Appendix — (DOC) [file pone.0250268.s001.doc]

**S1 Appendix**

The BILOG program, with which the 2PLM-MLE on Table 1 was produced, is presented in S1 Table.

***S1 Table BILOG program for the 2PLM-MLE producing for fixed item parameters***

| >GLOBAL DFName ='pattern10.dat',NPArm=2, LOGistic, SAVE;  >SAVE SCORE='2PL --test10.SCO';  >LENGTH NITems=(10);  >INPUT NTOtal=10,NIDchar=1;  >ITEMS;  >TEST1 TNAme='TEST0001',  INUmber=(1(1)10),  SLOpe=(1.000(0)5,0.800(0)5),  THReshld=(-4.0000(1.000000)0.0000,0.0000(1.000000)4.0000),  GUEss= (0.0000(0)10),  FIX =(1(0)10);  (1A1,10A1)  >CALIB ACCel=1.0000,NOSprior,RASch;  >SCORE METhod=1; |
| --- |

The BILOG program, with which the Biweight estimation on Table 1 was produced, is presented in S2 Table.

***S2 Table BILOG program for Biweight estimation, producing for fixed item parameters***

| >GLOBAL   DFName ='pattern.dat',NPArm=1, LOGistic, SAVE; >SAVE    SCORE='BIWEIGHT--test10.SCO'; >LENGTH   NITems=(10); >INPUT   NTOtal=10,NIDchar=1; >ITEMS; >TEST1  TNAme='TEST1',         INUmber=(1(1)10),         SLOpe=(1.000(0)5,0.800(0)5),         THReshld=(-4.0000(1.000000)0.0000,0.0000(1.000000)4.0000),         GUEss= (0.0000(0)10),        FIX =(1(0)10);         (1A1,10A1) >CALIB  ACCel=1.0000,NOSprior,RASch; >SCORE  METhod=1, BIWEIGHT; |
| --- |

The BILOG program, with which the3PLM-MLE on Table 1 was produced, is presented in S3 Table.

**S3 Table BILOG program for the 3PLM-MLE producing for fixed item parameters**

| >GLOBAL   DFName ='pattern.dat', NPArm=3, LOGistic, SAVE; >SAVE    SCORE='3PL-test10.SCO'; >LENGTH   NITems=(10); >INPUT   NTOtal=10,NIDchar=1; >ITEMS; >TEST1  TNAme='TEST1',         INUmber=(1(1)10),         SLOpe=(1.000(0)5, 0.800(0)5),         THReshld=(-4.0000(1.000000)0.0000, 0.0000(1.000000)4.0000),         GUEss= (0.2000(0)10),         FIX =(1(0)10);         (1A1,10A1) >CALIB  ACCel=1.0000, NOSprior, RASch; >SCORE  METhod=1; |
| --- |
