# Supplementary material for: The robust estimation of examinee ability based on the four-parameter logistic model when guessing and carelessness responses exist
Source: PLoS One. 2021 Apr 29;16(4):e0250268. doi: 10.1371/journal.pone.0250268 (PMC8084210; doi:10.1371/journal.pone.0250268)
Supplement: Attachment — Submitted filename: Response to Reviewers 3.25.doc [file pone.0250268.s004.doc]

Review Comments to the Author

Reviewer #3: The manuscript was revised and improved in many aspects, however the major changes required was not properly addressed. The major shortcoming of the manuscript is that the simulation study does not provide a fair comparison between the models. Using 3PLM-MLE and 4PLM-MLE the parameters c and γ was considered known, but it doesn’t match with the true generated data. For example, in simulation case 2, γ=1-p** for the 4PLM-Robust, which matches the true generated data, but it was set to γ=0.97 for 4PLM-MLE, regardless of the true generated data. Since c and γ are parameters that should be estimated, doesn’t seem to be fair to assume a wrong value to them when it’s possible to inform the true value. In the previous example, γ should be 1-p** for 4PLM-MLE.

 Response：If the true generated model follows exactly the 3PL-MLE or the 4PL-MLE in the test, the design and the aim of the manuscript will be complicated and blurry. And the first aim for the researchers will be a comparison between the two different generation models of guessing responses. Moreover, there is few common points between the two different generation models.

So it need the general consensus on **Guessing Principle** for all the researchers. As we know, if the examinee knows the answer to the item or is sure to answer correctly (for example, the probability is greater than 25%), then the examinee will not guess the answer of the item randomly. And more, random guessing is generated by the following model, Equation (12) (Mislevy & Bock, 1982):

$\text{P}_{vi}=\left\{\begin{matrix} 0.20if\psi(\theta_{v}-b_{i})\leq p* \\ \psi(\theta_{v}-b_{i})otherwise \end{matrix}\right.$ (12)

The generation model, Equation (12) tallies with the Guessing Principle.

The manuscript is very interesting in the sense the present the 4PLM-Robust model, which seems to be more flexible to accommodate many patterns of response disturbance. However, the article does not contain simulations to verify that it is robust enough when the disturbance pattern does not follow its assumptions. For example, a simulation case in which the true generated model follow exactly the 3PL-MLE or the 4PL-MLE to see the behavior of the 4PLM-Robust. Therefore, the main conclusion is that the best model is the one that corresponds to the true generated-response model, as should be expected.
#----------------------------------------------------------------------------------------------------------------------------------

Minor changes:
Introdution – There is no mention to the proposed method.

Response：In the new manuscript, “But these strategies still have faults and needs to be improved. So in the study, a robust estimation based on the 4PLM (4PLM-Robust)” is added for the Introdution sections. proposed to robustify the ability estimation.

Page 7 (lines 122-130) ,9 (line 180), 10 (lines 182, 201), 26 (line 446) – The equations are misconfigured.

Response：In the new manuscript, some sentences and some equations are revised. And more,

(1) $\sum_{i=1} (u_{i}-P(\eta_{i}))=0$ is revised as $\sum_{i} a_{i}\cdot(u_{i}-P(\eta_{i}))=0$ in the new manuscript;

(2) $\sum_{i} (u_{i}-P(\eta_{i}))\cdot(P(\eta_{i})-c_{i})/[P(\eta_{i})(1-c_{i})]=0$ is revised as $\sum_{i} a_{i}\cdot(u_{i}-P(\eta_{i}))\cdot(P(\eta_{i})-c_{i})/[P(\eta_{i})(1-c_{i})]=0$ in the new manuscript.

Line 198: donot = do not

Response：It is revised in the new manuscript.

Line 247: Replace smaller for larger ?

Response：It is revised in the new manuscript.

Line 250: Replace larger for smaller?

Response：It is revised in the new manuscript.

Line 257: “The response of Subject #6 most seriously deviates from the referenced pattern of subject #1”. Is not the opposite?

Response： The difficult parameter of the 10-*th* item is far larger than the ability of Subject #6, and the probability is very small. For expected probability, the score on the 10-*th* item of Subject #6 or Subject #1 should be 0 score, but the score of Subject #6 on the 10-*th* item is 1 in Table 1. So in the manuscript, “The score on the 10-*th* item of Subject #6 can be seen as most seriously deviated from the referenced pattern of subject #1, because on the probability it can be conclude that Subject #6 has a guessing response on the 10-*th* item”.
